# Comparative evaluation of target volumes defined by deformable and rigid registration of diagnostic PET/CT to planning CT in primary esophageal cancer

**DOI:** 10.1097/MD.0000000000005528

**Published:** 2017-01-10

**Authors:** Yanluan Guo, Jianbin Li, Peng Zhang, Qian Shao, Min Xu, Yankang Li

**Affiliations:** Department of Radiation Oncology, Shandong Cancer Hospital and Institute, Jinan, Shandong, China.

**Keywords:** deformable image registration, diagnostic PET/CT, esophageal cancer, planning CT

## Abstract

**Background:**

To evaluate the geometrical differences of target volumes propagated by deformable image registration (DIR) and rigid image registration (RIR) to assist target volume delineation between diagnostic Positron emission tomography/computed tomography (PET/CT) and planning CT for primary esophageal cancer (EC).

**Methods:**

Twenty-five patients with EC sequentially underwent a diagnostic ^18^F-fluorodeoxyglucose (^18^F-FDG) PET/CT scan and planning CT simulation. Only 19 patients with maximum standardized uptake value (SUV_max_) ≥ 2.0 of the primary volume were available. Gross tumor volumes (GTVs) were delineated using CT and PET display settings. The PET/CT images were then registered with planning CT using MIM software. Subsequently, the PET and CT contours were propagated by RIR and DIR to planning CT. The properties of these volumes were compared.

**Results:**

When GTV_CT_ delineated on CT of PET/CT after both RIR and DIR was compared with GTV contoured on planning CT, significant improvements using DIR were observed in the volume, displacements of the center of mass (COM) in the 3-dimensional (3D) direction, and Dice similarity coefficient (DSC) (*P* = 0.003; 0.006; 0.014). Although similar improvements were not observed for the same comparison using DIR for propagated PET contours from diagnostic PET/CT to planning CT (*P* > 0.05), for DSC and displacements of COM in the 3D direction of PET contours, the DIR resulted in the improved volume of a large percentage of patients (73.7%; 68.45%; 63.2%) compared with RIR. For diagnostic CT-based contours or PET contours at SUV_2.5_ propagated by DIR with planning CT, the DSC and displacements of COM in 3D directions in the distal segment were significantly improved compared to the upper and middle segments (*P* > 0.05).

**Conclusion:**

We observed a trend that deformable registration might improve the overlap for gross target volumes from diagnostic PET/CT to planning CT. The distal EC might benefit more from DIR.

## 1 Introduction

Radiotherapy, as one of the main effective and relatively safe treatment modalities, is now widely used in the treatment of esophageal cancer (EC).^[1]^ The efficiency of radiotherapy in EC is based on the accuracy and precise quantification of tumor variations and complete identification of the potential tumor tissue.^[2,3]^ Hence, it has become increasingly important to accurately delineate and define the target volume. Advances in imaging have made a profound impact on the definition of target volumes of cancer for radiotherapy treatment planning (RTP).

^18^F-fluorodeoxyglucose Positron emission tomography/computed tomography (^18^F-FDG PET/CT), which can significantly affect CT-based tumor contours by providing information on both biological and metabolic features of cancer, has demonstrated significant value in EC radiotherapy, for example, by identifying metastases to mediastinal lymph nodes.^[3]^ Metabolic information can be incorporated into the RTP process by performing a dedicated RTP PET/CT simulation with the patient positioned in the treatment position on a flat couch top.^[4]^ Several studies have demonstrated that adding PET to the RTP might significantly improve the accuracy of the contours of the tumor volumes and reduce variability when defined by different radiation oncologists.^[5]^

PET/CT data are primarily acquired for staging and diagnostic imaging studies, not radiation therapy (RT) treatment planning. Because of the significant costs and logistical problems involved, unfortunately, it is unlikely that many patients would undergo both staging and dedicated treatment planning PET/CT as part of their routine management. Thus, diagnostic PET/CT may be the only PET-based modality available to the radiation oncologist. Significant changes in patient position and anatomy between the diagnostic PET/CT and the planning CT highlight the need for advanced tools to aid in image registration for radiation therapy planning.

Image registration is the process of determining the point-by-point correspondence between 2 images so that the features in the images match. Rigid image registration (RIR) of the 2 CT images can effectively align the PET to the planning CT images to accurately define the volumes for radiation treatment.^[6]^ However, RIR, optimizing only translations and rotations, may not account for anatomic changes, including changes in patient positioning and soft tissue displacement due to breathing, peristaltic, cardiac, or involuntary motion. Deformable image registration (DIR) is an image processing technique with the potential to account for such changes.^[7]^ It maps the individual components (voxels) of one scan to those of another, thus attempting to resolve such differences. The correct estimation of these differences may permit the accurate transfer and propagation of radiotherapy target volume structures between image datasets. Moreover, the use of PET/CT for further dose escalation to PET-positive regions requires such acquisition transformation. The use of DIR has been shown to allow for more accurate registration of a PET/CT scan to an RTP CT scan in patients with head and neck tumors.^[6,8]^

Although many studies investigating the utility of DIR have been conducted, no information regarding the clinical impact of DIR in the target volume definition of PET/CT scans to planning CT scans for primary thoracic EC has been found in the literature. Therefore, in the present study, we evaluated the geometrical differences in target volumes propagated by DIR and RIR to assist in target volume definition between diagnostic PET/CT and planning CT in primary EC.

## 2 Patients and methods

### 2.1 Patient selection and characteristics

The ethics board of Shandong Cancer Hospital approved the study and every patient provided informed consent before enrolment into the study. From November 2013 to September 2014, we prospectively enrolled 25 patients with histologically proven EC (squamous) who were candidates for radiotherapy. The 25 patients were diagnosed with PET/CT. Excluded were patients with maximal SUV on PET of <2.0. The DIR process and the image after DIR were inspected by 2 specialists. Only those with no major misalignment between the CT and PET components were enrolled. In total, the image data from 19 patients (5 in upper thoracic EC, 9 in middle, 5 in distal) were available for analysis. The patient characteristics are listed in Table 1.

**Table 1:**
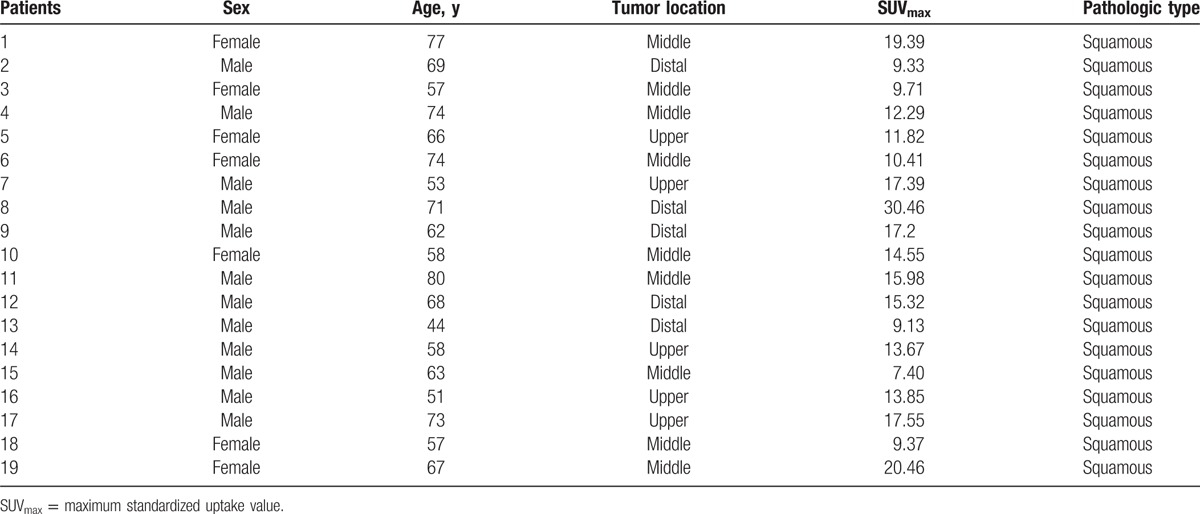
Characteristics of the patients enrolled in the study.

### 2.2 PET/CT image acquisition

Whole body PET/CT images were scanned 1 to 4 days prior to the planning CT scan as part of the routine diagnostic protocol for EC. All patients were asked to fast for at least 4 h before ^18^F-FDG PET/CT imaging. Each patient received 370 MBq (10 mCi) of ^18^F-FDG intravenously 40 min before scanning and rested in a supine position in a quiet and dimly lit room. All images were acquired with an integrated PET/CT scanner. Patients were positioned on a conventional diagnostic curved couch without the t-bar, with a pillow for head support. CT images without contrast (4.5-mm slices) were obtained during quiet breathing. With imaging times of 2 or 3 min per bed position, PET images were scatter-corrected and reconstructed by use of an ordered-subsets expectation maximization algorithm with a postreconstruction Gaussian filter at 5 mm full width at half maximum. The PET and CT images were imported into MIM software (MIM, 6.1.0, Cleveland, OH).

### 2.3 CT simulation and image acquisition

During the RTP planning CT simulation, all patients were immobilized using a thermoplastic mask. For each person, a contrast-enhanced planning CT scan of the thoracic region was performed under uncoached free breathing conditions on a 16-slice CT scanner (Philips Brilliance Bores CT, Cleveland, OH, USA)). For planning CT, each scan (360° rotation) took 1.0 s to acquire followed by a 1.8-s dead time with a 2.4-cm coverage. The 3-dimensional computed tomography (3DCT) scanning procedure took approximately 30 s. The planning CT images were reconstructed using a thickness of 3 mm and then transferred to MIM software.

### 2.4 Image registration algorithm

Deformable registration of images was performed with MIM Vista version 6.1.0 (MIM software), an intensity-based free-form deformable registration algorithm with essentially limitless degrees of freedom. This algorithm belongs to grayscale image-based algorithms and has been evaluated by Piper,^[9]^ who calculated the correlation of 2 CT images acquired weeks apart in the same patient after significant weight loss, determined the ability of the algorithm to recover the known target deformation, and measured the consistency of the algorithm. For recovery of the known target deformation, the mean residual errors were 10-fold smaller with deformable registration than with rigid registration. With the deformable registration algorithm, nearly 3/4 of the volume elements in the deformed target had a residual error of <1 mm relative to the standard of reference.^[9]^

### 2.5 Target volume delineation

The gross tumor volume (GTV) was first delineated on RTP planning CT images and the CT images of PET/CT datasets by the same radiation oncologist using a mediastinal window (window width = 400 HU, window level = 40 HU) setting, referred to as  
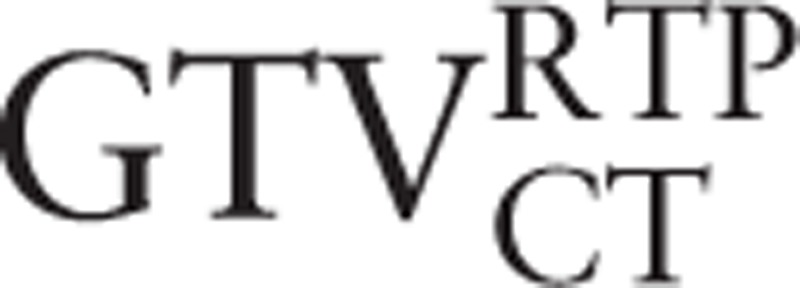
 and GTV_CT_. Given that no single optimal method of PET-based target volume delineation was used,^[10]^ the 3 PET-based contour delineation methods included:

(1) A manual PET contour (GTV_PETMAN_) was generated following delineation using a standardized window setting, with the window width equal to the maximum of the pixel intensity within the PET image and the window level equal to half this maximum.^[5]^
(2) Two PET contours were delineated using an absolute standardized uptake value (SUV) threshold of 2.5 (GTV_PET2.5_) and a threshold of 20% (GTV_PET20%_) of the maximum standardized uptake value (SUV_max_) within the target image.^[10–12]^

### 2.6 RIR and DIR of PET/CT scan to RTP planning CT scan

Given the low resolution of the PET images and the lack of clear discernible normal landmarks, all registration was undertaken using the CT components of the PET/CT and planning CT scans. Registration of the PET scans was therefore performed using the same registration parameters of the CT-to-CT based registration. For CT–CT registration, initially, a rigid registration focusing on the dorsal spine was performed automatically or manually using MIM software. Following rigid registration, deformable registration was then performed. In this process, the CT of the PET/CT scans was deformed to the CT of the RTP planning scan using the deformable registration algorithm described above.^[9]^ Because the automatic image registration algorithm considers the data from the entire image set, we placed a bounding box over the region of interest (ROI) to remove the influence of other parts of the body on the registration process. During the process, each PET voxel was mapped to a new position based on the transformations used in the CT–CT registration, resulting in a new PET/CT dataset that was deformably registered with the planning CT. In the process of registration, the GTV_CT_, GTV_PET20%_, GTV_PET2.5_, and GTV_PETMAN_ contours were transformed and propagated in 2 ways:

(a) A linear transformation and rotations in the rigid process, and the contours were transformed to  
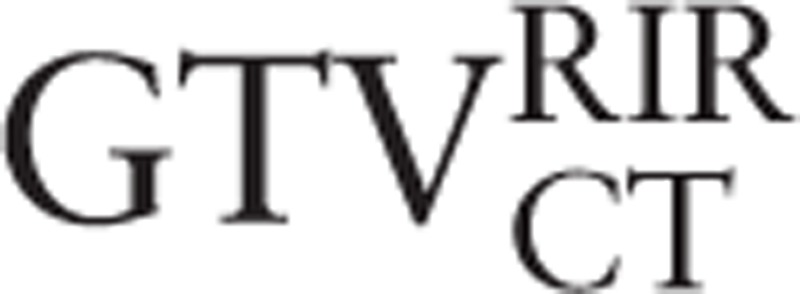
,  
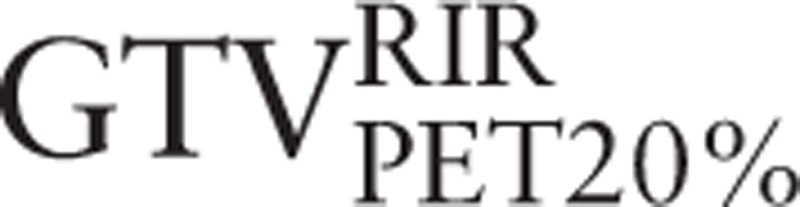
,  
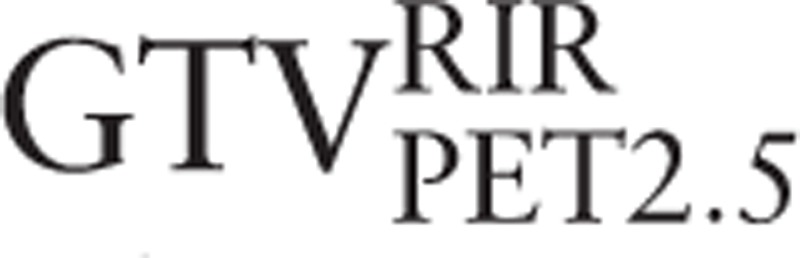
, and  
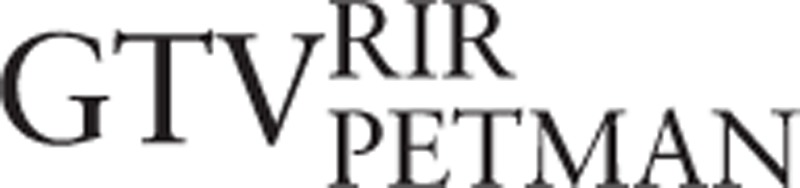
.
(b) The “warp” deformed and propagated at the RTP CT in the process of deformable registration. The radiation oncologist chose to adjust the deformed contours at the ROI's uninvolved barriers. The adjusted deformed contours were defined as  
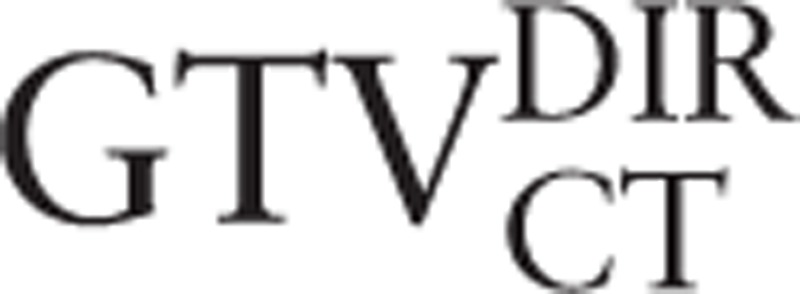
,  
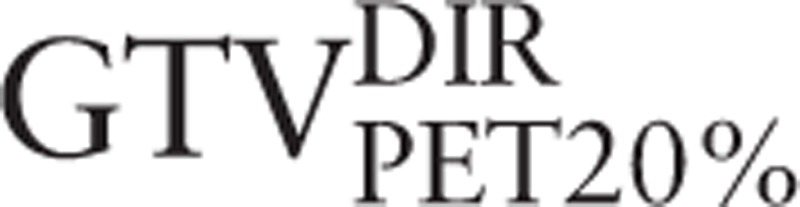
,  
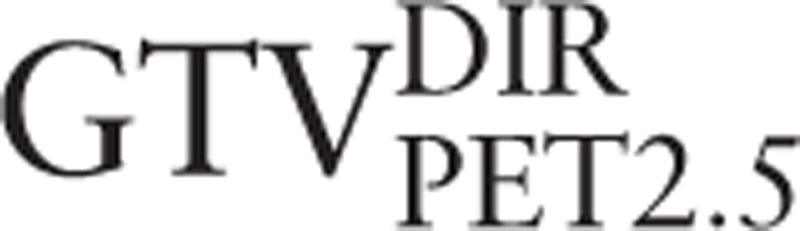
, and  
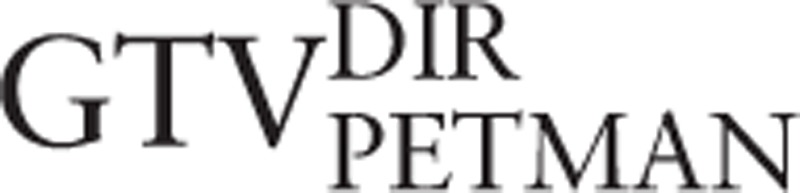
.

### 2.7 Displacements of the COM

To assess positional change, the displacements of COM in the left–right (LR), anterior–posterior (AP), and cranial–caudal (CC) planes between the GTV before and after each registration step and the GTV on the RTP scan were derived as Δx, Δy, and Δz, respectively. The 3D vector (the displacements of COM 3D direction distances) was calculated as follows: V = (Δx^2^ + Δy^2^ + Δz^2^)^1/2^. The 3D vector distance change ΔV% = |(V_DIR_ − V_RIR_)/V_RIR_|, V_DIR_ and V_RIR_ represent the 3D vector distance between GTV_DIR_ and GTV_RTP_ and GTV_RIR_ and GTV_RTP_, respectively.

### 2.8 Dice similarity coefficient

To assess volumetric shape and positional change between the GTV after each registration step and the same GTV on the RTP scan, the Dice similarity coefficient (DSC) was calculated. The DSC for the ratio of overlap between volumes A and B is described as follows: DSC = 2(A ∩ B)/(A + B).^[13]^ The DSC percentage change, ΔDSC% = |(DSC_DIR_ − DSC_RIR_)/DSC_RIR_%|. DSC_RIR_ and DSC_DIR_ represent the DSC of GTV_RIR_ and GTV_RTP_ and GTV_DIR_ and GTV_RTP_, respectively.

### 2.9 Statistical analysis

Statistical analysis was performed using the SPSS software package (SPSS 17.0). Mean ± standard deviation was used to represent the quantitative parameters. A paired sample *t* test was used to examine any differences between data pairs. One-way analysis of variance was used for pairwise comparison of ΔV% and ΔDSC% between different groups of EC patients. A value of *P* < 0.05 was regarded as significant.

## 3 Results

To investigate the correlations between different locations of EC and the change of DSC and displacements of COM in 3D directions, patients were divided into 3 groups: those with lesions located in the upper segment (5 patients) were in group A, middle segment lesions (9 patients) were in group B, while lesions in the distal segment (5 patients) were in group C.

### 3.1 DIR evaluation

Ideally, the deformed diagnostic CT would be identical to the planning CT image. In practice, it was similar but not identical due to imaging artifacts and spatial resolution inaccuracies. Inspection of the deformed regions suggests good agreement of the tumor and thorax after DIR. Meanwhile, visual inspection also revealed no major misalignment between the CT and PET components.

### 3.2 Variation of volumes

The GTVs on the PET/CT scan, after RIR and after DIR, and the GTVs as delineated on the RTP planning CT scan are listed in Table 2. The percentage volume change between  
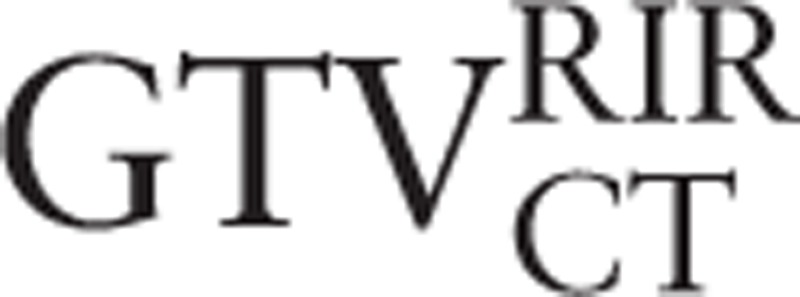
 and  
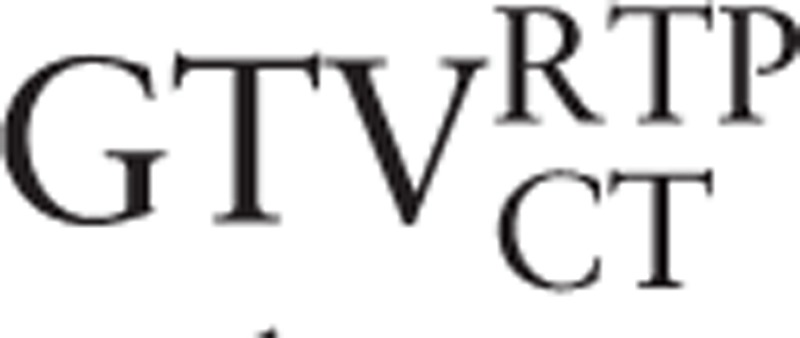
 was 10.57 ± 16.57%; this was reduced to a percentage volume change of 1.11 ± 14.48% between  
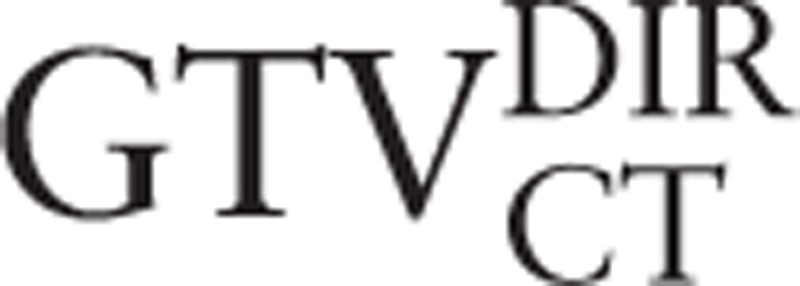
 and  
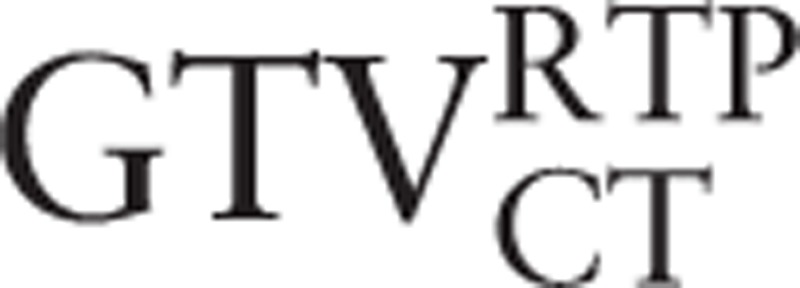
 following deformation, showing an improved approximation to the planning CT scan using DIR (*P* = 0.005). Similar improvements were not observed for the same comparison for PET contours (*P* = 0.081–0.919).

**Table 2:**
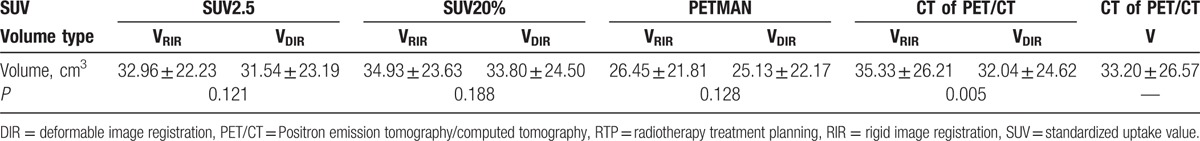
Gross tumor volume (cm^3^) (GTV) on PET/CT scan, after rigid registration and after deformable registration, and the GTV as delineated on the RTP planning CT (mean ± standard deviation) scan.

### 3.3 Displacements of COM in LR (ΔX), AP (ΔY), CC (ΔZ), and 3D directions

Table 3 lists the displacements of COM between the GTV contours on PET/CT scan when registered using both RIR and DIR with the RTP planning CT scan when compared with GTV obtained using the planning CT scan alone. The displacements of COM in LR and CC directions revealed no significant differences for any contours (*P* = 0.135–0.829). In the AP (ΔY) direction, they all showed statistical significance (*P* = 0.006–0.035). In the 3D direction, the significant reduction in the displacements of COM on GTV delineated on the CT of the PET/CT scan and the manual contours on the PET scan registered using DIR with the planning CT were observed (*P* = 0.006, 0.000), but no significant differences in the similar comparison were observed on PET contours at SUV_2.5_ and SUV_20%_ (*P* = 0.070, 0.597). However, for displacements of COM in the 3D direction of PET contours at SUV_2.5_ and SUV_20%_, DIR resulted in 73.7% (14/19) and 68.4% (13/19) of patients who improved compared with RIR, respectively.

**Table 3:**
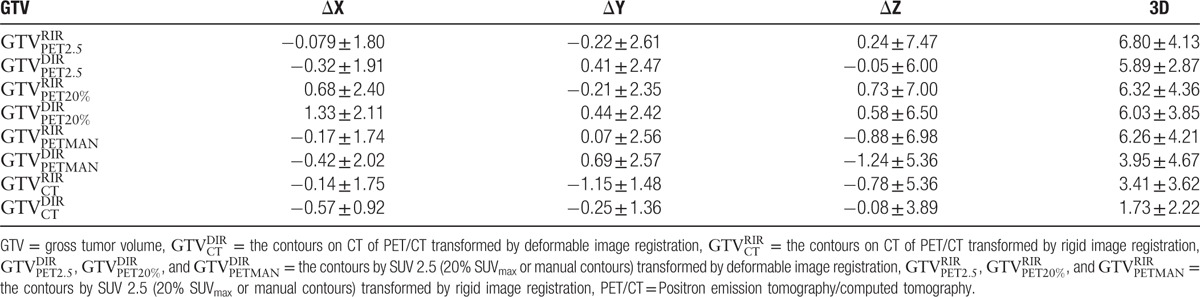
Displacements of the center of mass (mm) between the GTV contours on PET/CT scan when registered using both rigid and deformable approaches with the planning CT scan when compared with contours obtained using planning CT scan alone.

For group C, the displacements of COM in the 3D directions between  
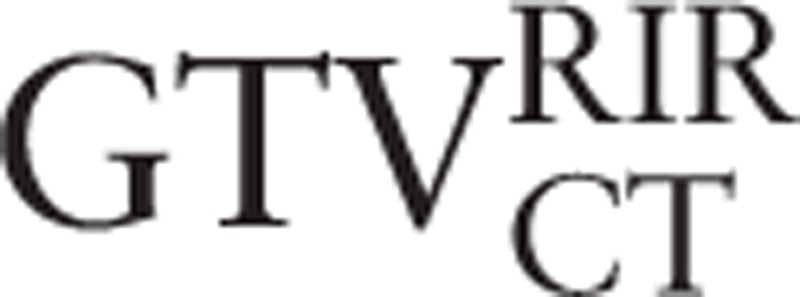
 and  
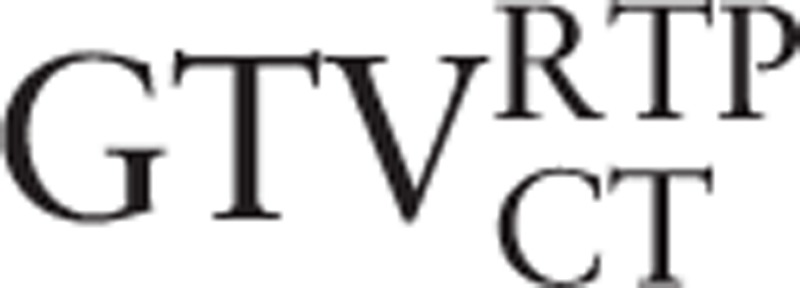
 or  
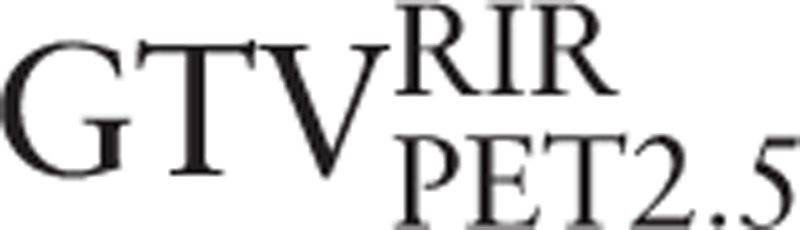
 and  
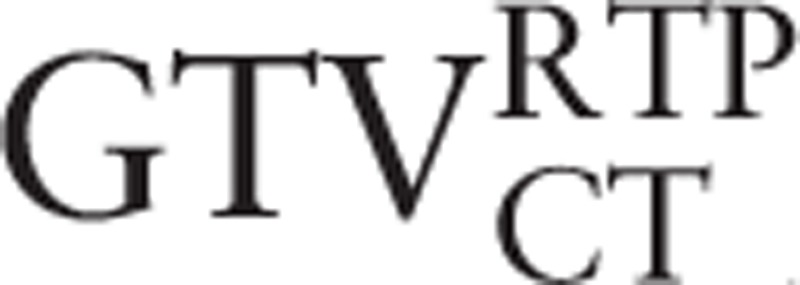
 were significantly larger than those of  
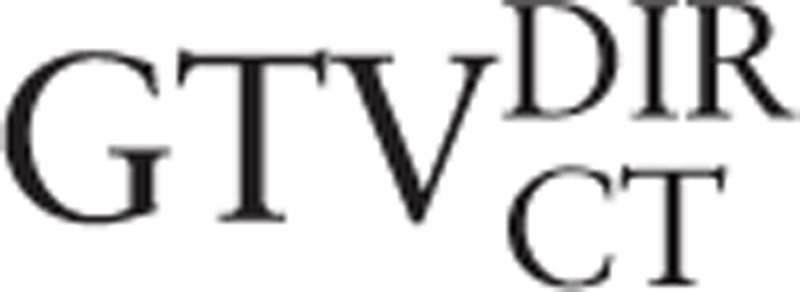
 and  
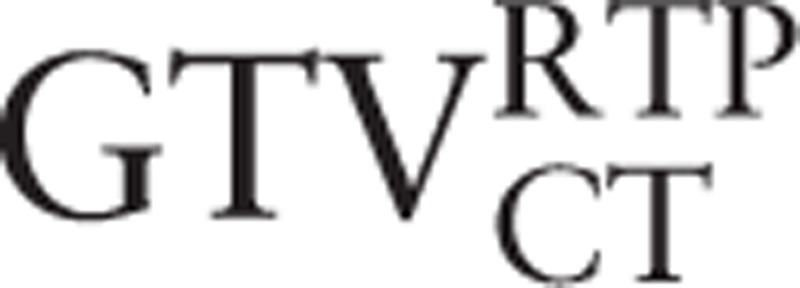
 or  
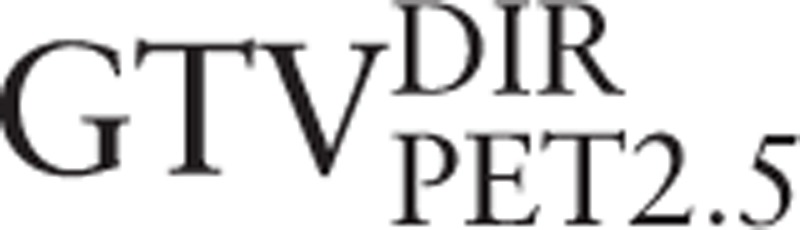
 and  
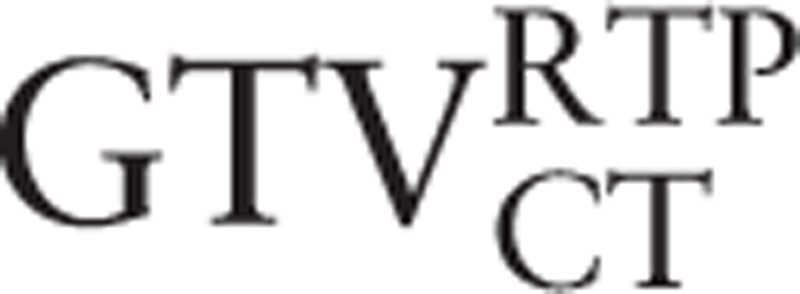
, respectively (*P* = 0.046, 0.048). Meanwhile, group C achieved smaller ΔV% than groups A and B (*P* = 0.046, 0.042, respectively). However, for PET contours at SUV_20%_ and manual contours, significant results were not observed for the similar comparisons (*P* > 0.05).

### 3.4 Variations of DSC

**Figure 1:**
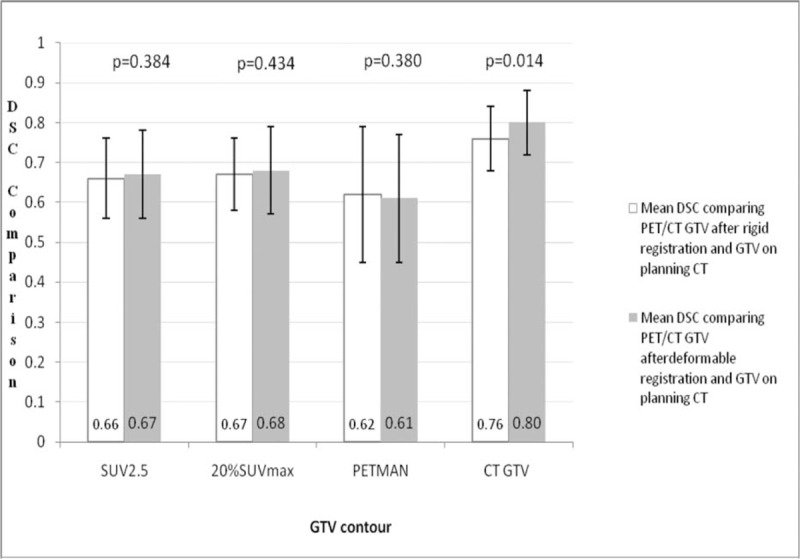
The DSC comparing the various GTVs after rigid image registration and deformable image registration with the same GTVs obtained on the planning CT scan. The range is denoted by the error bars, the mean values are shown, and the 2-tailed significance is listed above each comparison. CT = computed tomography, DSC = Dice similarity coefficient, GTV = gross tumor volumes.

The DSC comparing the various GTVs after RIR and DIR with the same GTVs obtained on the planning CT scan are summarized in Fig. 1. In the comparison of GTV_CT_ contours, the use of DIR resulted in a significant increase in DSC (*P* = 0.014). In marked contrast, all 3 PET-based contours revealed no similar improvements in registration with deformable over rigid registration (*P* = 0.380–0.434). However, for the DSC of PET contours at SUV_2.5_ and SUV_20%_, and manual contours, DIR resulted in 73.7% (14/19), 63.2% (12/19), and 63.2% (12/19) of patients who improved compared with RIR, respectively.

For group C, the DSC between  
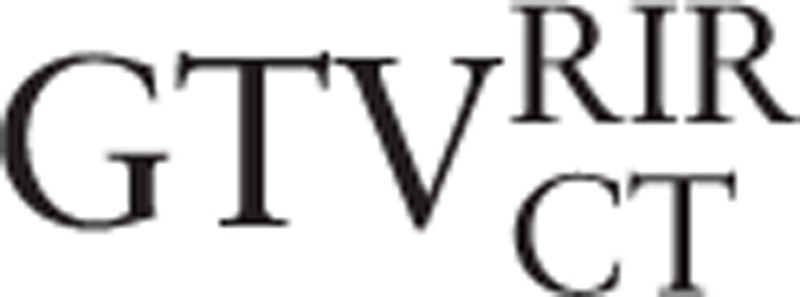
 and  
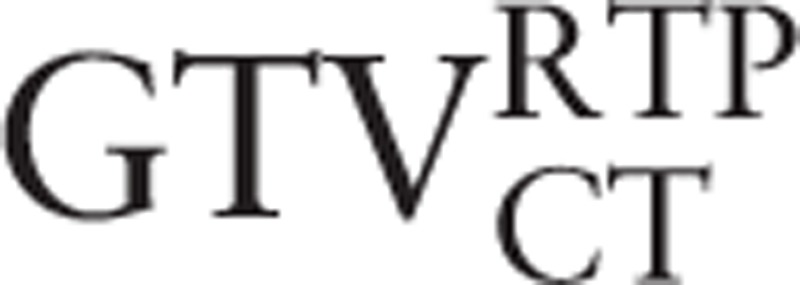
 or  
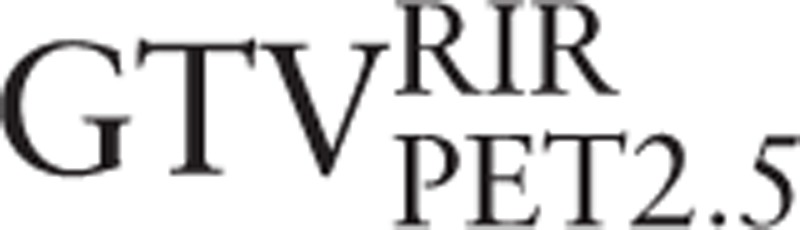
 and  
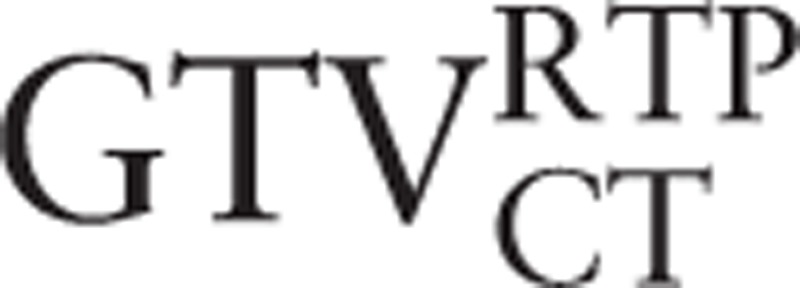
 was significantly larger than that of  
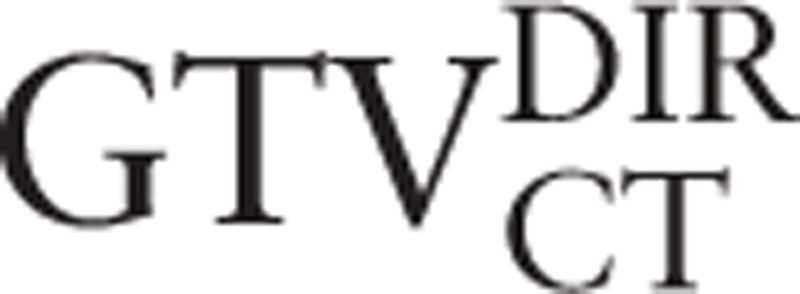
 and  
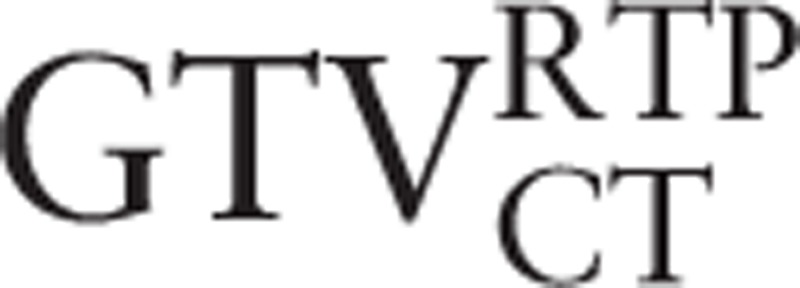
 or  
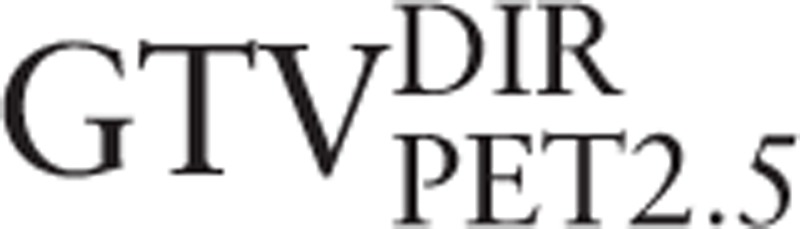
 and  
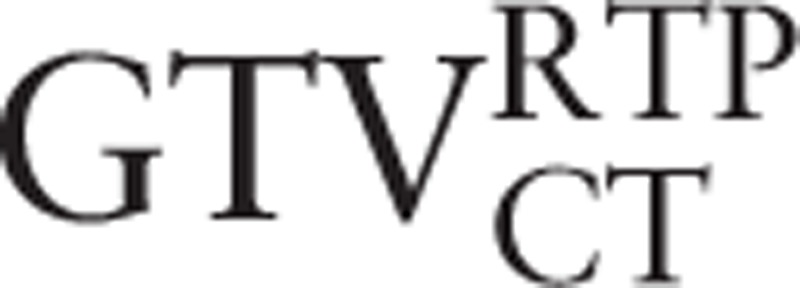
, respectively (*P* = 0.008, 0.042). Meanwhile, group C achieved smaller ΔV% than groups A and B (*P* = 0.016 and 0.015, respectively). However, for PET contours at SUV_20%_ and manual contours, significant results were not observed for the similar comparisons (*P* > 0.05).

## 4 Discussion

The performance and utility of DIR has already been investigated in many studies of head and neck cancer, breast cancer, and lung cancer patients. Kovalchuk et al integrated preoperative PET/CT deformed with postoperative treatment planning CT and a proved that it is a powerful tool for target volume delineation in head and neck patients undergoing postoperative intensity modulated radiation therapy (IMRT).^[14]^ DIR segmentation technique could be used for semiautomating ITV production from 4DCT for lung patients accurately and efficiently. Therefore, the ITVs automated-produced are immediately and clinically acceptable or requiring minimal editing. DIR also represents a significant time saving for clinicians.^[15]^ DIR was also applied to evaluate intrafraction variability and deformation of the lumpectomy cavity, breast, and nearby organs by Glide-Hurst et al, and large variability were observed between them.^[16]^ Despite these reports, few studies to date have evaluated the clinical impact of DIR in target volume definition of diagnostic PET/CT scan to planning CT for primary thoracic EC.

In this study, the results demonstrated that significant improvements using DIR were observed in volume size, displacements of COM in the 3D direction, and DSC between the comparison of  
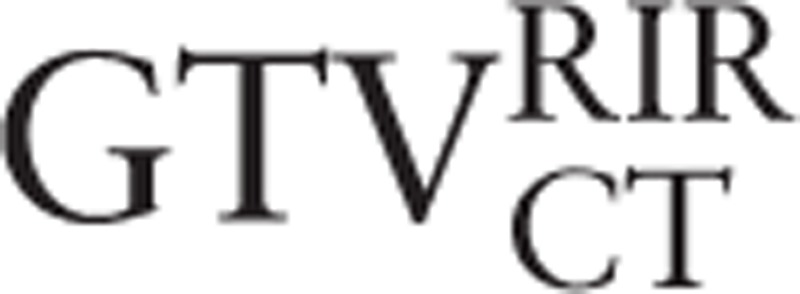
 with  
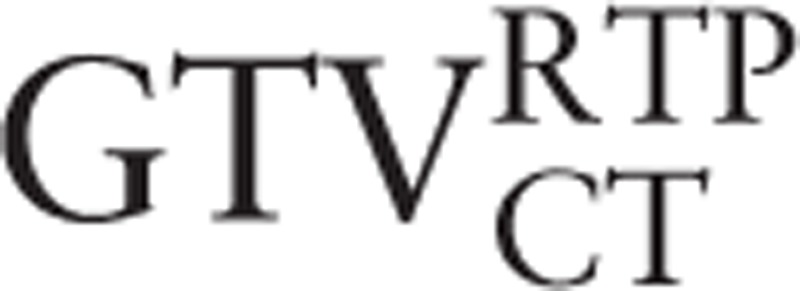
 and the comparison of  
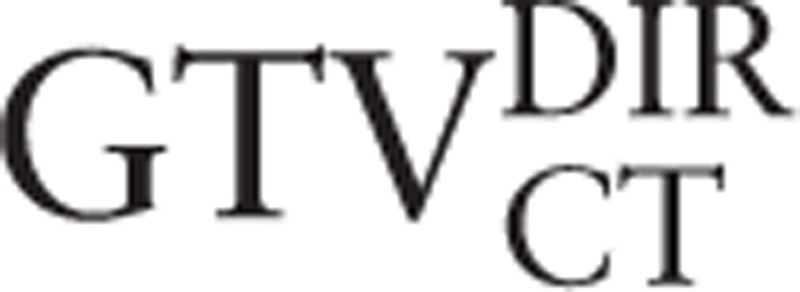
 with  
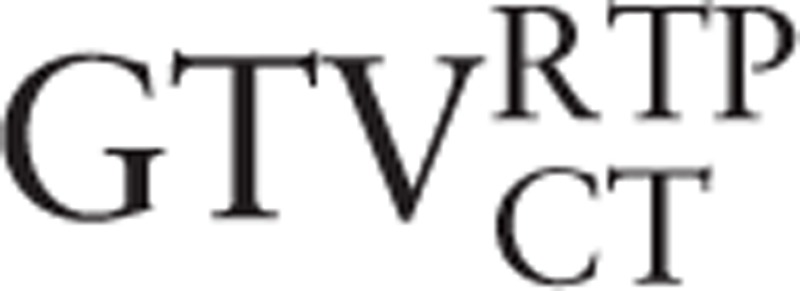
. In contrast, significant improvements were not observed using DIR for transferring and propagating PET contours from the PET/CT scan to the planning CT scan, using the CT-based registration as an intermediate step. However, for DSC and displacements of COM in the 3D direction of PET contours at SUV_2.5_, SUV_20%_, and manual contours, DIR resulted in the improved volume of a large percentage of patients (73.7%, 68.45%, 63.2%) compared with RIR. Although the results of CT-based and PET-based contours were inconsistent, it revealed a trend that DIR might improve the overlap for gross target volumes from diagnostic PET/CT scan to planning CT scan. Moreover, it also suggests that caution should be exercised when applying DIR for propagating gross target volumes between diagnostic PET/CT and planning CT for EC because of the inconsistent results. Our results were comparable to a previous investigation by Hanna et al.^[17]^ They compared the DSC and the displacements of COM between staging PET/CT rigid registered and deformable registered with RTP PET/CT scan for 10 lung cancer patients. The improvements were only observed for CT-to-CT-based contours with deformable registration. Ireland et al performed RIR and DIR between PET/CT and planning CT for 5 head and neck cancer patients by some anatomic landmarks,^[8]^ and they proved that nonrigid registration was more accurate than rigid registration. Furthermore, PET/CT scanned with patients in a standardized diagnostic position nonrigid registered with planning CT could provide images with greater accuracy than PET/CT scanned in a treatment position rigid registered with planning CT.

Among all subjects of multiple tumors, displacements of COM in LR and CC directions between the GTV contours on PET/CT scan when registered using both RIR and DIR with the planning CT scan, when compared with contours obtained using the planning CT scan, were not significantly different (*P* = 0.135–0.829). However, significant differences in the displacements of the AP direction were observed in the similar comparison (*P* = 0.006–0.035). Compared with the above results, in the investigation by Fortin et al, that there were consistent longitudinal differences in the location of the GTVs delineated on either rigidly or deformably registered PET/CT images.^[18]^ These observations may suggest that DIR was performed on a slice-by-slice basis, that is, no significant deformations might be performed longitudinally and LR laterally.

In this article, the results also indicated that for PET contours at SUV_2.5_ and diagnostic CT-based contours propagated from diagnostic PET/CT to planning CT in the distal segment, the DSC and displacements of COM in 3D directions improved more significantly than in the upper and middle segments after DIR. This does not allow us to draw a conclusion but it could implicate that DIR might be more valuable for distal EC. In practice, unlike in head and neck cancer, due to breathing, peristaltic, cardiac, or involuntary motion, the motion magnitude for tumors located in the distal segment was larger than that for tumors located in the upper and middle segments. In a retrospective study by Fortin et al of 10 lung and 10 head and neck cancer patients, 2 sets of GTVs based on either rigidly or deformably registered PET/CT (registered with planning PET/CT) scans were compared, the results indicated that unless significant anatomical differences between PET/CT and planning CT images are present, DIR was shown to be of marginal value when delineating GTV.^[19]^ This suggests that DIR might be more valuable for significant anatomical differences, such as distal EC, lower lobe lung tumors, and so on.

From these results, we could also suggest that the intensity-based registration algorithm performed in this study used voxel similarity measures such as the sum of square gray value differences, cross-correlation, local correlation, and mutual information between images that works best when the 2 sets of images have been acquired at the same resolution. Obviously, diagnostic CT and planning CT belong to the same modality images, and both of them are fast scanning images. PET provides functional information of gross target volume that is complementary to the GTV of anatomic information from modalities such as CT and magnetic resonance imaging. Additionally, registration of the PET scans was performed using the same registration parameters of the CT-to-CT-based registration. Hence, the CT-based contours presented significant deformations after DIR than PET contours. On the other hand, it should also be noted that the application of the image transformation derived from the CT-to-CT registration depends on the PET/CT being “inherently hardware registered.” However, this is not always true for PET/CT studies, in which the mismatch in temporal resolution (i.e., CT scans are acquired much faster than PET) can result in misalignment due to respiratory motion.^[20]^ This might be 1 reason for the inconsistent results between PET-based and CT-based contours. Thus, if registration of a staging PET scan is to be used as a means of incorporating PET in RTP simulation, 1 potential solution to overcome this issue is acquisition of the CT imaging with 4D information and subsequent registration of the staging PET scan to the 4DCT scan.

Dose painting is to deliver optimized dose redistribution according to the functional imaging information. The application of PET to radiotherapy provides new channels to the clinical application of dose painting to further improve tumor control.^[21]^ Diagnostic PET/CT might be the only PET-based modality available to the radiation therapist for dose painting. The size and shape of the subvolume is a crucial factor which confirms the level of the escalated dose; therefore, the delineation method used for contouring is vital for the applicable maximum dose. This study might be a valuable guide for target volume propagating for DIR dose painting treatment planning (especially dose painting by contours) in EC.

One limitation of this study is that planning contrast-enhanced CT with the patient lying immobilized in thermoplastic mask fixation was first used in this investigation. This may have an impact on the accuracy of the image registration as contrast would not normally be used for the attenuation correction CT acquired for PET. It may be possible to alleviate this problem by changing the density on the contrast-enhanced CT.^[22]^ In addition, in this investigation, some target volumes were manually delineated; possible intraclinician contouring variation between the scan sets may have led to variable comparisons between the CT and PET components although all delineation was performed by the same observer using published guidelines.^[23]^

## 5 Conclusions

Deformable registration might improve the overlap for gross target volumes from the diagnostic PET/CT scan to the planning CT scan. This also suggests that caution should be exercised when applying DIR for propagating target tumors between diagnostic PET/CT and planning CT for EC. The distal EC might benefit more from DIR.
